# Hyperuricemia and Cardiovascular Risk

**DOI:** 10.7759/cureus.14855

**Published:** 2021-05-05

**Authors:** Lauren Shahin, Komal M Patel, Milad K Heydari, Marc M Kesselman

**Affiliations:** 1 Rheumatology, Dr. Kiran C. Patel College of Osteopathic Medicine, Nova Southeastern University, Davie, USA

**Keywords:** hyperuricemia, gout, cardiovascular disease

## Abstract

As the prevalence of hyperuricemia (elevated uric acid levels in the blood) increases, the relationship between serum uric acid levels and cardiovascular risk has garnered increased interest. Several studies have highlighted that elevated uric acid levels are likely tied to increased cardiovascular disease risk. Specifically, the presence of hyperuricemia is well-established to contribute to the onset of gout (an inflammatory condition characterized by painful/swollen joints). Several studies have shown that the risk of developing gout is strongly associated with the degree of hyperuricemia. In this review, we will provide insight into the association between gout and cardiovascular disease risk. It is also important to gain insight into the pathophysiology of gout to understand the contributions to cardiovascular disease risk as well as improve diagnosis and target treatment more effectively. An interdisciplinary approach for gout management and areas for further investigation will be discussed in this review.

## Introduction and background

Gout is considered the most common type of inflammatory arthritis in the United States and is most prevalent among men [1]. This type of arthritis results from an inflammatory response to monosodium urate crystal deposition in and around the joints, most commonly affecting the big toe but crystal deposits may also develop in joints around the hands and feet. An analysis by Singh and colleagues demonstrated that the total number of individuals with self-reported gout increased from 8.3 million to 9.2 million over a period of eight years [2]. Gout has also been shown to increase with age, and patients frequently present with multiple co-morbidities including hypertension and diabetes [3]. Several risk factors contribute to the development of gouts, such as increased age, diet, and genetic predisposition [4].

The build-up of uric acid from the breakdown of purine nucleotides underlies the development of gout. Increased uric acid concentrations may result from both an increased production of uric acid and a decreased excretion of uric acid [1]. Although hyperuricemia (serum urate concentration levels greater than 6.8 mg/dL) is a necessary pathogenic factor for the development of gout, not all individuals with hyperuricemia develop clinical manifestations of gout (inflammatory arthritis, tophaceous deposits, and uric acid nephrolithiasis) [3].

There is emerging evidence of hyperuricemia in patients with uncontrolled gout playing a role in contributing to cardiometabolic comorbidities [5]. Studies have shown that increases in serum uric acid levels (which can lead to hyperuricemia and potentially contribute to gout) may be tied to an increased risk of cardiovascular disease and mortality [4]. Both gout and cardiovascular disease are associated with systemic inflammation and oxidative stress, which accelerates atherosclerosis resulting in the morbid-mortal outcomes of cardiovascular disease [6]. In addition, hyperuricemia has been shown to be associated with endothelial dysfunction as well as the oxidation of lipoproteins within atherosclerotic plaques (contributors to cardiovascular disease risk) [6]. 

This review aims to assess the interrelationship between gout and cardiovascular disease risk. It is also important to gain insight into the pathophysiology of gout to understand the contributions to cardiovascular disease risk as well as improve diagnosis and target treatment more effectively. An interdisciplinary approach for gout management and areas for further investigation will be discussed in this review.

## Review

It is well understood that a relationship exists between elevated uric acid levels and the risk of cardiovascular disease. Most recently, several studies have highlighted the role of uric acid as an independent biomarker of cardiovascular disease risk [7]. Several cardiovascular conditions including hypertension, coronary artery disease (CAD), cerebrovascular disease, vascular dementia, and pre-eclampsia have been associated with high uric acid levels. While hyperuricemia is common in many cardiovascular diseases as well as in post-menopausal women and those with renal disease and/or hypertension that can be associated with an increased risk of cardiovascular disease, multiple studies have demonstrated that hyperuricemia independently increases the risk of cardiovascular disease [7]. In a prospective study conducted in Rotterdam, an association between baseline serum uric acid levels and myocardial infarction was confirmed in the 4385 participants without a history of stroke or CAD [8]. In the Italian Progetto Ipertenione Umbria Monitoraggio Ambulatoriale (PIUMA) study, researchers found that the highest serum uric acid levels were significant predictors of cardiovascular events and death in hypertensive patients who were enrolled [9]. Similarly, the results of the Pressioni Arteriose Monitorate E Loro Associiazoni (PAMELA) study showed a significant increase in the risk of cardiovascular death for every 1 mg/dL increase in serum uric acid levels [4].

Hyperuricemia and hypertension

Several epidemiological studies have described an association between elevated serum uric acid levels and hypertension. In 1994, the Olivetti Heart Study in Southern Italy conducted a longitudinal epidemiological investigation evaluating the association between serum uric acid and hypertension in a sample of male workers in Southern Italy whose information on coronary heart disease risk factors was available at baseline and 12 years follow-up [10]. The results after multiple logistic regression analyses showed an independent, positive association between uric acid levels and the development of hypertension, even after adjustment for age, body mass index (BMI), serum total cholesterol, and triglycerides [10]. Another review and meta-analysis in 2011 found an association between hyperuricemia and incident hypertension with an adjusted risk ratio of 1.41 and 95% confidence interval of 1.23-1.59 [11]. For every 1 mg/dL increase in uric acid levels, the risk ratio for incident hypertension was 1.13 [11]. These effects were more pronounced in younger individuals and women [11].

Several other studies have identified hyperuricemia as an independent risk factor for hypertension. According to Kuwabara and colleagues, approximately 25% to 40% of untreated hypertensive patients have concomitant hyperuricemia [12]. The researchers found that patients with hypertension often exhibited hyperuricemia even if they were not taking any antihypertensive medications that elevated serum uric acid levels. In a cross-sectional study conducted by Kuwabara, the analysis showed that an increase in serum uric acid levels by 1 mg/dL increased the prevalence of hypertension by 1.2 fold even when adjustments were made according to age, BMI, dyslipidemia, diabetes, smoking, and estimated glomerular filtration rate [7]. These studies emphasized the importance of monitoring serum uric acid levels in hypertensive patients regardless of whether they are taking antihypertensive medication.

Hyperuricemia appears to play a role in the development of primary hypertension in adolescents. One study showed elevated uric acid levels (>5.5 mg/dL) in nearly 90 percent of adolescents with essential hypertension [13]. Uric acid levels were significantly lower in control and teens with either white coat or secondary hypertension [13]. In order to prevent hypertension, hyperuricemia at a young age needs to be addressed.

An elevated serum uric acid level has been strongly associated with elevated blood pressure [14]. Early animal studies provide evidence that elevated uric acid levels may lead to hypertension. In a study examining the effects of mild hyperuricemia in rats, the rats were treated with uricase inhibitors and it was found that their blood pressure increased as the uric acid levels increased. In this study, blood pressure was directly correlated with serum uric acid levels. When a xanthine oxidase inhibitor or uricosuric agent was used the uric acid levels were decreased [14]. These animal studies have shown that hyperuricemic hypertension induces vasoconstriction by activation of the renin-angiotensin-aldosterone system. This causes uric acid uptake into vascular smooth muscle cells leading to cellular proliferation and secondary arteriolosclerosis that impairs pressure natriuresis. Clinical research has also shown that correlations between the higher serum uric acid levels and calcification of the coronary artery, the decreased flow-mediated dilation, and higher pulse wave velocity, suggest that hyperuricemia is associated with arteriosclerosis [7]. 

Hyperuricemia and atherosclerosis

The link between hyperuricemia and the risk of atherosclerotic cardiovascular and cerebrovascular disease has been well-established [13]. In an observational cohort study testing the association between hyperuricemia and coronary artery calcification, the results showed that hyperuricemia is an independent risk factor for sub-clinical atherosclerosis in young adults [15]. Summarized data by Kanbay and colleagues suggests that hyperuricemia may cause atherosclerosis in the macrovascular beds such as the coronaries and the carotids as well as the microvascular damage in the renal vascular beds and may exacerbate vascular disease [16]. In a cross-sectional study of middle-aged adults, the investigators found that elevated serum uric acid levels were independently associated with the prevalence of atherosclerotic vulnerable carotid plaque [17].

Depending on the microenvironment, uric acid may act as an antioxidant or an oxidant [18]. Under ischemic conditions, xanthine oxidase uses oxygen as an electron acceptor instead of nicotinamide adenine dinucleotide (NAD+) resulting in the formation of superoxide anion and hydrogen peroxide. Oxidants cause endothelial dysfunction by reacting with and removing nitric oxide (NO), which prevents vasodilation of the endothelium. This promotes a pro-inflammatory state that causes endothelial dysfunction and contributes to atherosclerosis and cardiovascular disease [19]. Uric acid can also induce vascular smooth muscle cell proliferation in vitro by producing pro-inflammatory, pro-oxidative, and vasoconstrictive substances [16]. Uric acid stimulates the production of monocyte chemoattractant protein-1 (MCP-1), a chemokine involved in atherosclerosis. Increased production of MCP-1 increases cell proliferation and production of pro-inflammatory mediators [20].

Hyperuricemia and heart failure

Increased uric acid levels have been associated with oxidative stress, endothelial dysfunction, inflammation, and cardiovascular events [21]. Several studies have identified hyperuricemia as an independent unfavorable prognostic factor in heart failure patients. In the Cardiovascular Health Study, the incidence of heart failure was 21% in participants with chronic hyperuricemia and 18% in those without chronic hyperuricemia. The results showed that an increase of 1 mg/dL in serum uric acid levels conferred a 12% increase in the risk of new-onset heart failure [22]. In a recent analysis by the National Health and Nutrition Examination Survey (NHANES), the survey demonstrated the prevalence of hyperuricemia to be approximately 50% in patients with heart failure [23]. Among patients with heart failure, elevated serum uric acid levels and increased oxidative stress have been associated with increased mortality [24]. In a study on the prognosis of heart failure patients, for every 1 mg/dL increase in serum uric acid, the risk of all-cause mortality and the composite endpoint in heart failure increased by four percent and 28%, respectively [4].

In heart failure and other pathological conditions, anaerobic metabolism in tissues due to low oxygen availability increases the levels of serum lactic acid. As a result, the increased lactic acid level intensifies the reabsorption of uric acid in the kidney leading to an increase in serum uric acid levels [7]. According to another pathogenic hypothesis, serum uric acid functionally up-regulates the xanthine oxidase enzyme, which converts hypoxanthine to xanthine and xanthine to uric acid. The reactive oxygen species derived from xanthine oxidase may lead to cardiac hypertrophy, myocardial fibrosis, and left ventricular remodeling and contractility impairment [4].

Patients with heart failure and a history of gout represent a high-risk population. Using xanthine oxidase inhibitors as therapeutic agents for hyperuricemic patients with heart failure has gained interest. Allopurinol, a xanthine oxidase inhibitor currently used to treat gout, maybe a novel therapeutic agent for hyperuricemic patients with heart failure. Meanwhile, the efficacy of xanthine oxidase inhibitors on heart failure complicated by hyperuricemia remains uncertain and further investigation is needed. In a recent cohort study, continuous allopurinol use reduced heart failure readmissions or death in patients with a history of gout by 69% [24]. This protective association with allopurinol among heart failure patients and gout indicates that the effects of allopurinol may only be evident in patients with high xanthine oxidase activity. In an analysis by Bredemeier and colleagues, the findings showed that purine-like xanthine oxidase inhibitors may reduce the incidence of adverse cardiovascular outcomes, but higher doses of allopurinol may be associated with loss of cardiovascular protection [25]. There is still a lot of variability in the results of the clinical trials and further investigation on the effects of allopurinol must be conducted. In the Nihon University working group study of febuxostat and usual allopurinol therapy for patients with hyperuricemia (NU-FLASH) trial, 141 cardiac surgery patients with hyperuricemia and renal dysfunction were randomized to a febuxostat or allopurinol. The study showed that febuxostat reduced uric acid earlier than allopurinol and had superior anti-oxidant and anti-inflammatory effects [26]. More information is needed on the clinical benefit of using xanthine oxidase inhibitors for the treatment of patients with hyperuricemia and heart failure.

Pathophysiology of gout

The association between hyperuricemia, gout, and cardiovascular disease risk is based on the underlying pathophysiology of gout. A key pathological mechanism of gout is hyperuricemia, which is caused by enzyme dysfunction in the metabolism and elimination of uric acid [1]. Hyperuricemia usually occurs due to either overproduction of uric acid or under excretion [4].

**Figure 1:**
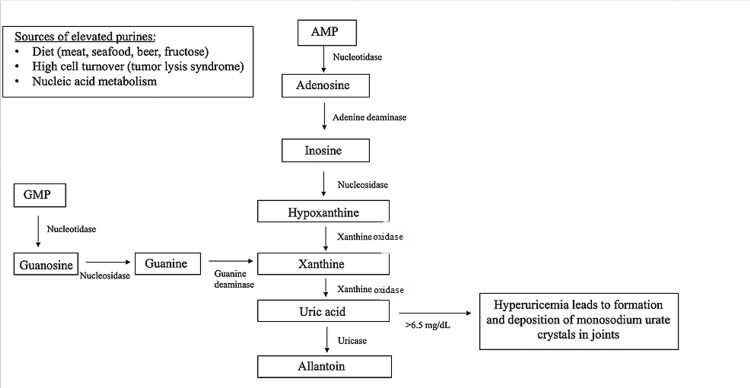
Purine metabolism leading to the production of uric acid and inflammatory response.

Deficiency of the enzymes involved in purine metabolism leads to hyperuricemia, as is characteristic of gout [1]. The metabolic pathway contributing to uric acid formation is presented in Figure 1. One mechanism that leads to hyperuricemia is an unbalanced ratio of purines [1]. Purines (purine nucleotides) are essential precursors for nucleic acid synthesis and also play important roles in metabolic signaling and coenzyme synthesis. Increased purine production during hypermetabolic states results in increased uric acid production (purine metabolism) [1]. Diet can also play a key role in the development of hyperuricemia. Specifically, meat, seafood, foods high in fructose and/or sodium, and alcohol, may increase uric acid concentrations, while foods rich in vitamin C, low-fat dairy products, and plant oils may reduce the risk of hyperuricemia and gout [1].

Another mechanism that results in hyperuricemia is decreased uric acid excretion through the kidneys [1]. Most of the uric acid load is eliminated by the kidneys and excreted in the urine, which can become impaired, leading to hyperuricemia [1]. The urate transporters on the proximal convoluted tubule of the kidney play an important role in regulating the serum uric acid levels via active transport [1]. The adenosine triphosphate (ATP)-binding cassette transporter G2 (ABCG2) also plays an important role in uric acid excretion in the kidney and the gut [27]. Dysfunction in the ABCG2 transporter leads to decreased excretion of uric acid through the gut resulting in a renal overload type of hyperuricemia [27]. Moreover, humans do not have the ability to convert uric acid to allantoin (uricase enzyme), which is a more soluble compound that can be excreted more efficiently [3]. Unlike uric acid, allantoin does not accumulate in crystals and is excreted unchanged through urine [3].

The presence of hyperuricemia can lead to the development of gout, which produces an inflammatory infiltrate involving neutrophils, macrophages, mast cells, and lymphocytes [1]. This process leads to an inflammatory response that results in swelling, erythema, and pain in the affected joint [1]. The deposition of uric acid crystals in the joint cavity initiates the inflammatory process leading to gout. Macrophages initiate the response by engulfing the uric acid crystals and are then capable of endothelial activation and releasing tumor necrosis factor (TNF), interleukin-1 (IL-1), interleukin-6 (IL-6), and interleukin-8 (IL-8) [1]. Intracellular monosodium urate crystals are recognized by the NACHT domain, leucine-rich repeat, and PYD-containing protein 3 (NLRP3) inflammasome, which is a multi-protein complex that activates the downstream innate immune response. The NLRP3 inflammasome activates caspase-1, which in turn, cleaves and activates IL-1b amplifying the inflammatory cascade [28]. Interactions between the monosodium urate crystals and toll-like receptors on the cell surface leads to the activation of nuclear factor kappa B activation and the transcription of pro-inflammatory cytokines [28]. After the initiation of the inflammatory process, local vasodilation stimulates neutrophil chemotaxis. Pro-inflammatory cytokines lead to vasodilation, with increased blood flow and permeability. Several chemotactic factors mainly involving IL-8 result in neutrophil activation. Neutrophils then interact with the monosodium urate crystals via bound inactivated C3b (iC3b) and immunoglobulin G (IgG) leading to the release of reactive oxygen species, prostaglandin E2, antimicrobial peptides, lysosomal enzymes, and pro-inflammatory cytokines [29]. Mast cells are also involved in gout and increase vascular permeability and vasodilation by producing histamine and IL-1. Both the classical and alternative complement pathways are also activated by monosodium urate crystals and amplify the inflammatory response. Gout is usually self-limiting and resolves after seven to 10 days, even without therapy [30]. Macrophages help clear the acute attack by phagocytizing the crystals and suppressing the inflammatory response via the release of transforming growth factor-beta (TGF-β).

Persistent untreated hyperuricemia may lead to a chronic gout condition, which is characterized by the presence of gouty tophi and structural joint diseases, such as bone erosion and cartilage damage [1]. The tophus is formed by a chronic granulomatous inflammatory response to monosodium urate crystals involving both cells of the innate and adaptive immune system [1]. Most tophi present as painless uninflamed subcutaneous nodules. Chronic inflammation in the synovium results in cartilage damage due to the stimulation of chondrocytes, which produce cytokines, nitric oxide, and matrix metalloproteases [1]. Chronic gout leads to bony erosions through the activation of nuclear factor kappa B (NF-kB) and the receptor activator of nuclear factor kappa-B (RANK) and RANK ligand (RANK-RANKL) pathway [1]. The RANK-RANKL pathway is important in bone remodeling in physiological and inflammatory conditions [31]. RANKL expressed in osteoblasts binds to its receptor RANK on the surface of osteoclasts promoting osteoclast activation. The RANKL-RANK interaction activates NF-kB, which is a transcription factor that up-regulates the expression of many other genes involved in the inflammatory process and inflammatory osteolysis. In a review on the pathogenesis of bone erosions in gout, Schlesinger and Thiele describe how monosodium urate crystals seem to alter the RANKL-osteoprotegerin balance indirectly promoting osteoclastogenesis in patients with gout [31]. Osteoblasts release pro-inflammatory cytokines leading to erosions and bone destruction [1]. Osteoclast precursors are also abundant within the synovial fluid in gout and at the tophus bone interface. RANKL is expressed by T cells in the tophus and enhances osteoclast formation. The imbalance in osteoblast and osteoclast function results in bone remodeling and resorption. 

Management and treatment

There is overwhelming evidence that patients with gout have an increased risk of cardiovascular disease, and by targeting the underlying mechanisms of gout with targeted approaches and management, the risk of cardiovascular disease can be reduced. Management of gout patients by an interdisciplinary team of primary care physicians, rheumatologists, and cardiologists using a comprehensive approach might improve the overall health outcomes of these patients. In a pilot study assessing the feasibility of a multi-disciplinary team-based program for the education and monitoring of gout patients, Fields and colleagues found that a multi-disciplinary team approach consisting of rheumatologists, pharmacists, trained registered nurse (RN) gout educators, and social workers improved the value of care for gout patients [32]. The results of the study showed that about 80% of the subjects felt that their nurse-educator experience made a positive impact on their management and 50% felt that the pharmacist calls were helpful. Overall, the researchers found that this multi-disciplinary approach was feasible and suggested further evaluation of this strategy to improve the quality of life of gout patients [32].

The first-line treatment for acute gout flares includes non-steroidal anti-inflammatory drugs (NSAIDs), steroids, and colchicine. Colchicine is an anti-mitotic drug that inhibits monosodium urate crystal activation of the NLRP3 inflammasome, blocks the release of IL-1b, and suppresses the expression of cell regulation genes at micromolar concentrations [33]. Microtubules mediate the assembly of the NLRP3 inflammasome leading to the activation and propagation of the inflammatory cascade. Colchicine inhibits tubulin polymerization and attenuates macrophage NLRP3 inflammasome activation [34]. It has also been used as an anti-inflammatory treatment in cardiovascular disease. Some studies have shown a lower incidence of cardiovascular complications in gout patients treated with colchicine [35]. Meanwhile, another trial showed that short-term low-dose colchicine did not improve endothelial function in patients with coronary artery disease [35]. However, an exploratory analysis showed that endothelial function was significantly improved in the subgroup of patients with leukocyte activation [36]. Further research on the effects of colchicine on cardiovascular risk in gout patients is needed.

For patients with gout refractory to NSAIDs and colchicine, the FDA has approved the use of an anti-IL-1b antibody, canakinumab, for gout inflammation. Recent studies showed that a single dose of canakinumab during an acute flare provided rapid and effective pain relief and prolonged suppression of flares and inflammation in patients with gout [37]. In a randomized, double-blind trial testing the effects of three doses of canakinumab on patients with a history of myocardial infarction and a high sensitivity C-reactive protein level, the results showed that canakinumab significantly reduced C-reactive protein levels and that anti-inflammatory therapy along with the 150 mg dose of canakinumab lowered the incidence of recurrent cardiovascular events compared to the placebo group with a hazard ratio of 0.85 [38].

In patients diagnosed with gout, the goal is to reduce the serum urate levels using urate-lowering drugs such as xanthine oxidase inhibitors, uricosurics, or a recombinant uricase [35]. Xanthine oxidase inhibitors have antioxidant properties, which reduce the production of reactive oxygen species that result from purine metabolism. In a review on xanthine oxidase inhibitors for the prevention of cardiovascular events, Bredemeier and colleagues emphasize that oxidative stress is an important factor that may be implicated in the pathogenesis of hypertension and heart failure [25]. The results of a meta-analysis study concluded that allopurinol (less than or equal to 300 mg) decreases blood pressure and creatinine levels in patients with hyperuricemia with or without antihypertensive treatment [39]. However, there is still a lot of contradictory evidence regarding the possible cardiovascular effect of xanthine oxidase inhibitors and further studies are needed to determine the mechanisms by which these effects occur [25]. Some anti-hypertensive drugs such as β-blockers, thiazides, and loop diuretics have been shown to lower the renal excretion of uric acid and aggravate hyperuricemia [35]. On the other hand, statins and fenofibrate increase renal urate excretion [35]. A case-control study determining the independent association of antihypertensive drugs with the risk of incident gout among people with hypertension suggests that calcium channel blockers and losartan may be protective against the risk of gout among people with hypertension [40]. In a paper addressing targeted therapy in gout, Perez-Ruiz and colleagues recommend a minimum target for serum uric acid of 6 mg/dL for all gout patients [41].

In a recent cohort trial conducted by Plein and colleagues, the researchers evaluated whether patients with early rheumatoid arthritis have cardiovascular disease that is modifiable with disease-modifying antirheumatic drug therapy (DMARD) [42]. The patients were randomized to either an etanercept and methotrexate group or to an initial methotrexate treat to target strategy group. The results showed improved vascular stiffness and patients actively receiving disease-modifying antirheumatic drugs (DMARDs) experienced lower cardiovascular event risk than those who discontinued [42]. The data from this study imply that immune-modulating treatment may be personalized in patients with rheumatoid arthritis to improve joint-specific outcomes and cardiovascular disease. This study highlights the importance of identifying patients at the earliest stage of rheumatoid arthritis. Similar to rheumatoid arthritis patients, early intervention with gout patients may help improve health outcomes especially since crystal deposition and subclinical inflammation precede the clinical onset of gout [43]. 

A cross-sectional study exploring the association between serum uric acid and cardiovascular disease in rheumatoid arthritis patients discovered that serum uric acid levels were also higher in patients with rheumatoid arthritis and cardiovascular disease [44]. The clinical use of serum uric acid as an early biomarker of future cardiovascular disease events in gout and rheumatoid arthritis patients needs to be further investigated.

When evaluating cardiovascular involvement in patients with autoimmune rheumatoid diseases, Markousis-Mavrogenis and colleagues demonstrated that cardiovascular magnetic resonance (CMR) is an ideal diagnostic tool [45]. CMR allows for early identification of cardiovascular disease and can assess both cardiac function and characterize myocardial tissues in relation to edema and fibrosis [45]. The researchers recommend the use of CMR to evaluate patients with acute rheumatic diseases before the patient presents with clinical manifestations of heart disease. Because patients with gout also have a risk of cardiovascular disease, further investigation on the use of CMR in gout patients should be considered.

## Conclusions

Based on the findings of all the epidemiological studies, it is evident that an elevated serum uric acid level is an independent risk factor for cardiovascular disease and hypertension. Although there has been an increased interest in the association between hyperuricemia and cardiovascular disease, further studies are needed to provide a more holistic understanding of the functions of uric acid in cardiovascular disease. It is also important to emphasize that urate-lowering drugs such as xanthine oxidase inhibitors are currently not indicated for asymptomatic hyperuricemia and further investigation on these drugs as potential therapeutic agents needs to be performed. Since there is insufficient data to support any specific therapeutic agent for patients with hyperuricemia and cardiovascular disease, screening high-risk patients early is of utmost importance. Education is also imperative to increase awareness about gout and improve adherence to therapy to ensure optimal treatment of patients, and reduce the risk of adverse cardiovascular outcomes. Implementing an early screening, management, and treating to target approach will hopefully prevent the onset of heart disease and improve survival outcomes for gout patients.
